# RETRACTION: Clinical Efficacy of Ablative Laser Combined With Pulsed Dye laser in the Treatment of Pathological Scars: A Systematic Review and Meta‐Analysis

**DOI:** 10.1111/iwj.70433

**Published:** 2025-03-26

## Abstract

**RETRACTION:** TianY. , LiM. , ChengR. , YuanJ. , and Haol. , “Clinical Efficacy of Ablative Laser Combined With Pulsed Dye laser in the Treatment of Pathological Scars: A Systematic Review and Meta‐Analysis,” International Wound Journal 21, no. 2 (2024): e14567, 10.1111/iwj.14567.PMC1082852042052984

The above article, published online on 30 January 2024, in Wiley Online Library (http://onlinelibrary.wiley.com/), has been retracted by agreement between the journal Editor in Chief, Professor Keith Harding; and John Wiley & Sons Ltd. Following an investigation by the publisher, all parties have concluded that this article was accepted solely on the basis of a compromised peer review process. The editors have therefore decided to retract the article. The authors did not respond to our notice regarding the retraction.

**RETRACTION:** Y. Tian , M. Li , R. Cheng , J. Yuan , and l. Hao , “Clinical Efficacy of Ablative Laser Combined With Pulsed Dye laser in the Treatment of Pathological Scars: A Systematic Review and Meta‐Analysis,” International Wound Journal 21, no. 2 (2024): e14567, 10.1111/iwj.14567.PMC1082852042052984

The above article, published online on 30 January 2024, in Wiley Online Library (http://onlinelibrary.wiley.com/), has been retracted by agreement between the journal Editor in Chief, Professor Keith Harding; and John Wiley & Sons Ltd. Following an investigation by the publisher, all parties have concluded that this article was accepted solely on the basis of a compromised peer review process. The editors have therefore decided to retract the article. The authors did not respond to our notice regarding the retraction.

